# A world without bacterial meningitis: how genomic epidemiology can inform vaccination strategy

**DOI:** 10.12688/f1000research.13793.1

**Published:** 2018-03-27

**Authors:** Charlene M.C. Rodrigues, Martin C.J. Maiden

**Affiliations:** 1 Department of Zoology, University of Oxford, Peter Medawar Building for Pathogen Research, Oxford, UK

**Keywords:** Bacterial meningitis, meningococcal disease, Neisseria meningitidis, genomic epidemiology, vaccine

## Abstract

Bacterial meningitis remains an important cause of global morbidity and mortality. Although effective vaccinations exist and are being increasingly used worldwide, bacterial diversity threatens their impact and the ultimate goal of eliminating the disease. Through genomic epidemiology, we can appreciate bacterial population structure and its consequences for transmission dynamics, virulence, antimicrobial resistance, and development of new vaccines. Here, we review what we have learned through genomic epidemiological studies, following the rapid implementation of whole genome sequencing that can help to optimise preventative strategies for bacterial meningitis.

## Introduction

**Figure 1.**
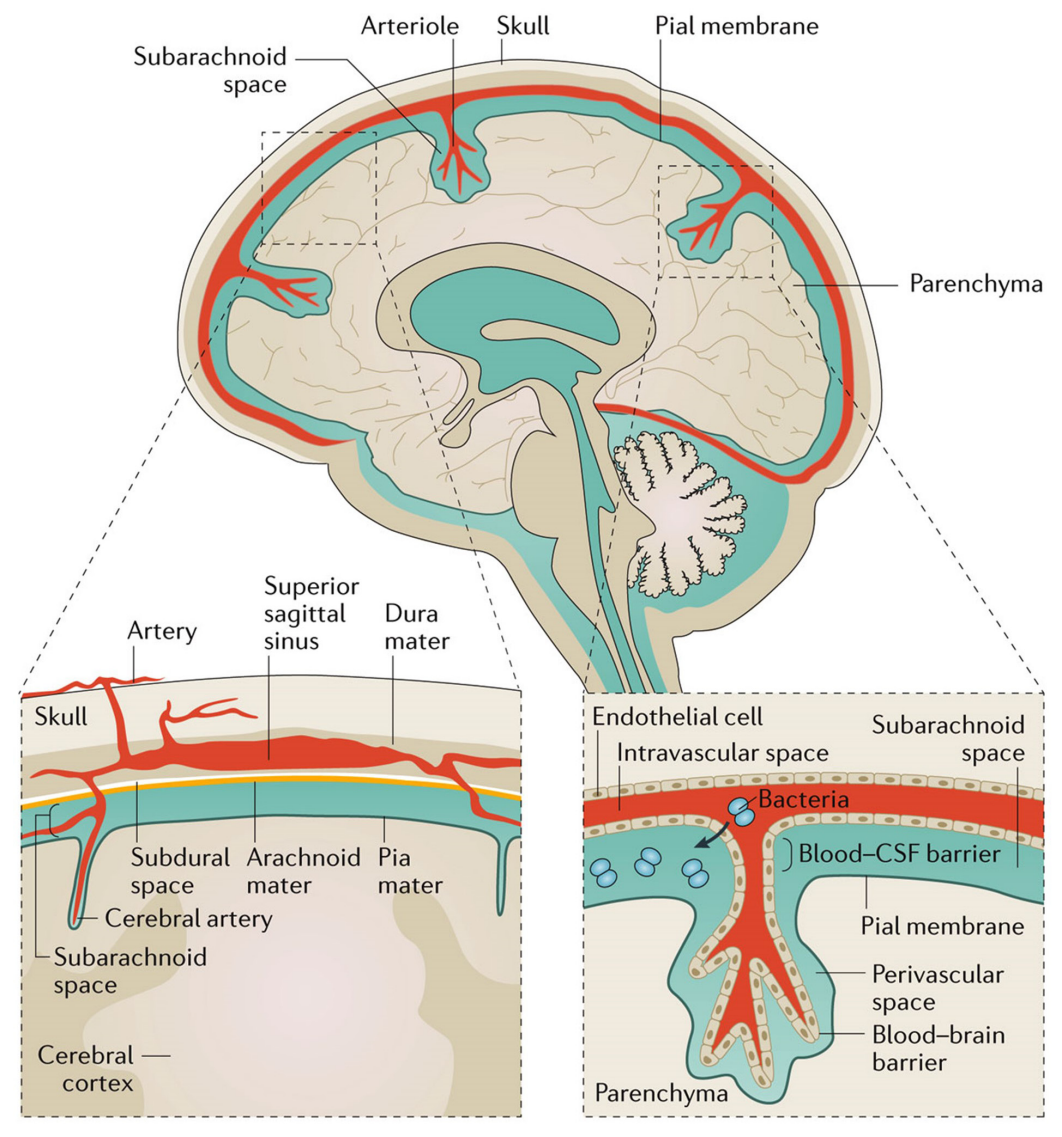
Anatomical representation of the human brain and meningeal structures affected by bacterial meningitis. The bacteria access the subarachnoid space from the blood, crossing the blood-brain barrier. Here, they replicate and cause inflammation as the host attempts to control the developing infection. The ensuing inflammation and ongoing infection result in major morbidity and mortality. CSF, cerebrospinal fluid. Figure reproduced unchanged with permission ^4^.

Bacterial meningitis describes infection of the subarachnoid space with bacterial pathogens, resulting in inflammation of the brain linings (meninges), a condition that causes significant morbidity and mortality worldwide. Bacteria reach the subarachnoid space through haematogenous or direct contiguous spread, where they replicate with resultant local meningeal inflammation and potential for involvement of the brain tissue ( Figure 1). In addition, where haematogenous spread of bacteria causes meningitis, persistence of bacteria in the blood, septicaemia, can rapidly develop into multi-organ failure and death.

Depending on age, geographic location, immune system function, and vaccine implementation, the incidence rates and causative organisms of bacterial meningitis differ ^1, 2^. In 2013, there were an estimated 303,500 deaths globally from meningitis, attributed to *Streptococcus pneumoniae* (n = 79,100), *Neisseria meningitidis* (n = 65,700), *Haemophilus influenzae* type b (Hib) (n = 64,400) and other agents (n = 94,200) ^3^. Despite highly effective vaccination programmes against the major pathogens, disease persists. This review will discuss what we have learned through genomic epidemiological studies, from elucidating transmission networks to describing bacterial biodiversity, with the aim of improving the use of existing vaccines and novel vaccine development.

## Global burden of bacterial meningitis

Bacterial meningitis in newborns in the first seven days of life is most commonly caused by group B streptococcus ( *Streptococcus agalactiae*) and *Escherichia coli* through vertical transmission from the birth canal and perineal region. After the first week of life, cases are mainly nosocomial or acquired via horizontal transmission, and *Listeria monocytogenes* and *S. pneumoniae* also contribute to disease burden ^5, 6^. Vaccination against Hib, *N. meningitidis*, and *S. pneumoniae* has greatly altered the epidemiology of bacterial meningitis in older children and adults over the last three decades ( Table 1) *. H. influenzae* meningitis is now extremely rare in countries with high uptake of the conjugate polysaccharide (Hib) vaccine, but cases can occur in unvaccinated individuals or with non-b serotypes. *S. pneumoniae* and *N. meningitidis* cause most disease, with meningococcus predominating in older children and adolescents, and pneumococcus predominating in adults. Other causes include: *L. monocytogenes* in the elderly or immunocompromised; *Staphylococcus aureus* co-existent with endocarditis; and *H. influenzae* co-existent with otitis media or sinusitis ^7, 8^.

**Table 1.** Characteristics of the three main pathogens that cause bacterial meningitis worldwide. Hib, *Haemophilus influenzae* type b; PCR, polymerase chain reaction; PCV, pneumococcal conjugate vaccine. Information from chapters 8, 14, and 17 of *Epidemiology and Prevention of Vaccine-Preventable Diseases* ^17^.

	*Haemophilus influenzae*	*Streptococcus pneumoniae*	*Neisseria meningitidis*
**Bacterial** **features**	Gram negative coccobacillus. Six capsular types recognised (a–f). Type b (Hib) predominated in invasive disease. Since vaccination began in 1990, non-b types have been causing disease.	Gram positive cocci in chains. Over 90 serotypes based on capsular polysaccharides identified. Prevalence varies by region, with 10 serotypes responsible for 62% of disease.	Gram negative diplococcus. 12 recognised polysaccharide capsular groups. Disease caused predominantly by 6 serogroups (A, B, C, W, X, Y).
**Carriage**	Carried in the nasopharynx as part of the normal commensal. Prior to vaccination, Hib was carried predominantly by young children.	Carried in the nasopharynx as part of the normal commensal. Varies by age and geographical distribution, but ranges 5–60%.	Carried in the nasopharynx as part of the normal commensal. Varies by age and geographical distribution, but ranges from 1–40%.
**Disease**	Invasive disease with Hib manifests with meningitis, epiglottitis, pneumonia, septic arthritis, cellulitis, osteomyelitis and septicaemia. Non-b type disease can cause similar infections, increasingly noted in Europe.	Invasive disease in upper and lower respiratory tract (mastoiditis, pneumonia), central nervous system (meningitis, cerebral abscess) and septicaemia and localised upper respiratory tract (otitis, sinusitis).	Invasive meningococcal disease comprising septicaemia and meningitis can affect all age groups. Early disease is non-specific and deterioration of clinical condition can occur very rapidly.
**Diagnostic** **methods**	Isolation from a sterile site by culture (chocolate agar). Serotyping with either slide-agglutination or serotype-specific PCR.	Isolation of *S. pneumoniae* from a normally sterile site – by culture or PCR. Serological testing using antibody reactivity to capsular polysaccharides for determination of serotype.	Isolation of *N. meningitidis* from a normally sterile site – by culture or meningococcal PCR ( *ctrA*). Serological testing using antibody reactivity to capsular polysaccharides for determination of serogroup.
**Age group** **affected**	Prior to vaccination, Hib was the leading cause of bacterial meningitis in children under 5 years.	Young children and the elderly at highest risk of invasive disease.	Infants and under 5 year olds at highest risk, adolescents have increased risk compared to other adults. Epidemics can affect all age groups in susceptible populations.
**Global** **epidemiology**	Disease occurs sporadically worldwide, but the use of Hib has greatly reduced the incidence. Hib vaccine has been introduced in 190 countries since 1990, with global infant coverage of 70% in 2017.	Disease occurs sporadically worldwide, with invasive pneumococcal disease amongst the main causes of global mortality through pneumonia, meningitis and sepsis deaths. Introduction of pneumococcal vaccines has had a major impact on the disease caused by these serotypes, but non-vaccine serotypes still cause disease.	Occurs as both epidemic and endemic disease, variable on geographic region. The “Meningitis Belt” in sub-Saharan Africa had cyclical epidemics prior to introduction of conjugate polysaccharide A vaccine. Industrialised countries (Europe, Australasia, North America) have low incidence endemic disease. Outbreaks have been associated with mass gatherings e.g. university students, military recruits, Hajj pilgrims; resulting in targeted vaccinations of high risk groups.
**Morbidity-** **meningitis**	Hearing impairment, neurological sequelae in 15–30%.	Hearing impairment, neurological sequelae.	Psychological, cognitive or physical sequelae in 30%.
**Mortality-** **meningitis**	3–6%	8% in children, 22% in adults.	5–17%
**Vaccines** **available**	Conjugate polysaccharide capsular type b vaccine. No vaccines are available against non-b strains.	Pneumococcal conjugate vaccine - 7 valent (PCV7) 4, 6B, 9V, 14, 18C, 19F and 23F. PCV10 - 1, 4, 5, 6B, 7F, 9V, 14, 18C, 19F and 23F. PCV13 - 1, 3, 4, 5, 6A, 6B, 7F, 9V, 14, 18C, 19A, 19F and 23F. Pneumococcal polysaccharide vaccine - 23 valent (PPV23) 1, 2, 3, 4, 5, 6B, 7F, 8, 9N, 9V, 10A, 11A, 12F, 14, 15B, 17F, 18C, 19F, 19A, 20, 22F, 23F, and 33F.	Conjugate polysaccharide vaccines against A, C, W, Y (monovalent or quadrivalent). Pentavalent conjugate vaccines including serogroup X are in development. Multi-peptide vaccines (4CMenB and recombinant lipoprotein rLP2086) against serogroup B. Plain polysaccharide vaccines against A, C, W, Y.

## Bacterial diversity: carriage and immune selection

With the exception of *L. monocytogenes*, the bacteria principally responsible for causing meningitis are carried asymptomatically as members of a healthy microbiota. Group B streptococcus is found in the vaginal tract of up to 20% of women, *E. coli* is found universally in the gut, *S. aureus* on the skin, and *S. pneumoniae*, *N. meningitidis*, and *H. influenzae* in the naso/oropharynx. There are likely to be interactions between the host immune system and the microbiota, although these are not fully understood, that result in the structured diversity observed in bacterial populations ^9^. This diversity is found amongst microorganisms of the same species, manifested as distinctive lineages (organisms that share a common ancestor and therefore exhibit genetic similarity) which persist through time. Even within these lineages, bacteria vary genotypically (that is, in their genetic constitution) and phenotypically (that is, in their observable characteristics) with an extensive capacity to alter protein expression states through phase variation. Thus, to appreciate the degree and mechanisms by which these populations are structured, it is necessary to study genome-wide variation among representative bacterial isolates. Only by understanding the host bacterial population structure can we start to identify the strains (bacteria that have a similar genotype and phenotype) that cause disease.

## Persistence of bacterial meningitis despite vaccination

**Figure 2.**
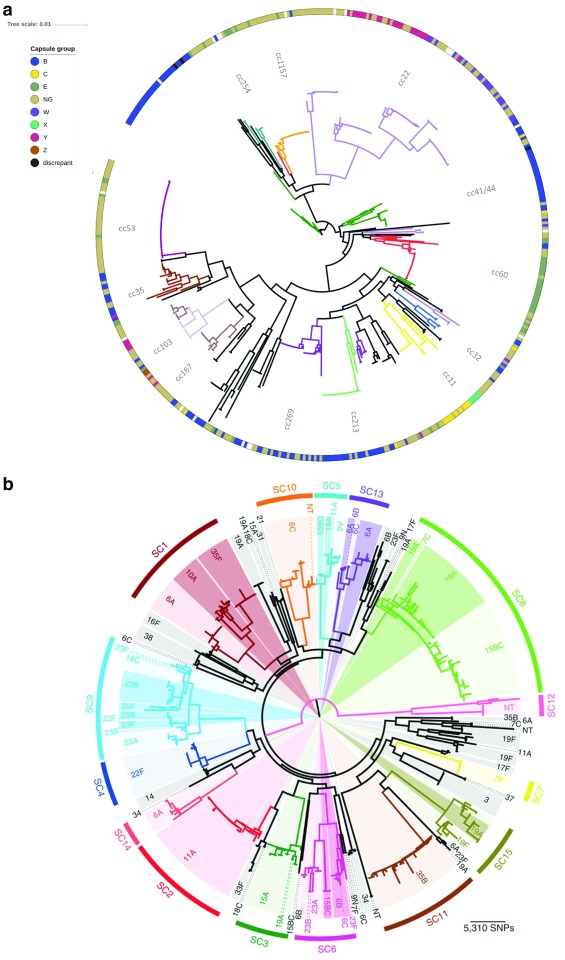
Population structure of *Neisseria meningitidis* and *Streptococcus pneumoniae* carriage isolates, demonstrating the diversity of genotype and capsular types. ( **a**) Allele-based phylogeny of 498 *N. meningitidis* carriage isolates from the UK obtained in 1999, generated by using seven multilocus sequence typing loci. Genotypes, described as clonal complexes (ccs), are shown by coloured clades on the tree branches. Capsular group is displayed on the peripheral band, data were derived from serological typing and genotyping, and “discrepant” isolates had non-concordant results. Phylogeny is visualised by using Interactive Tree of Life software ^18^. ( **b**) Maximum likelihood phylogeny of 616 *S. pneumoniae* carriage isolates from the US from 2001 and 2007, generated by using 106,196 polymorphic sites within 1,194 core genes. Monophyletic sequence clusters are shown and labelled peripherally. Within each sequence cluster, differential shading represents the different serotypes. SNP, single-nucleotide polymorphism. Figure reproduced unchanged with permission ^19^.

The diversity of *S. pneumoniae* and *N. meningitidis* challenges the continued success of vaccines and the elimination of bacterial meningitis caused by these organisms. Both bacteria exhibit high rates of horizontal genetic transfer (HGT) and comprise distinct, non-overlapping genetic lineages with varying degrees of pathogenicity. Each genetic lineage, recognised by multilocus sequence typing (MLST) as groups of sequence types (STs) called clonal complexes (ccs), can exhibit a variety of polysaccharide capsular types and undergo capsule switching ( Figure 2). Until 2013, all licenced vaccines for the prevention of bacterial meningitis pathogens were based on polysaccharide capsular antigens, key virulence factors as both acapsulate streptococci and meningococci very rarely cause invasive disease. Prevention of disease by capsular group was beneficial but allowed bacteria from hyperinvasive lineages that switched capsule to persist through carriage and ongoing transmission between hosts.

The first pneumococcal polysaccharide conjugate vaccine included seven capsular serotypes (PCV7) and subsequently increased to PCV10 and PCV13 with further iterations in development ( Table 1). This was based on the serotypes most frequently causing disease, but some capsular types were antigenically related, resulting in a degree of cross-protection. With more than 90 serotypes identified worldwide, the development of a universal vaccine remains challenging. In contrast, for meningococci there are only 12 recognised capsular groups, of which six serogroups cause almost all disease ( Table 1). Conjugate vaccines against serogroups A, C, W, and Y are available but not universally used ^10^. Until 2013, there was no licensed vaccine against serogroup B, a major cause of meningitis in industrialised countries. Hence, non-vaccine types continue to be carried in the host nasopharynx and transmitted, potentially causing disease in susceptible populations. Furthermore, through the extensive HGT in these pathogens, newly emerging hyperinvasive genotypes can arise. The introduction of a novel antigenic combination can result in epidemic or hyperepidemic disease.

## Genomic epidemiology

**Figure 3.**
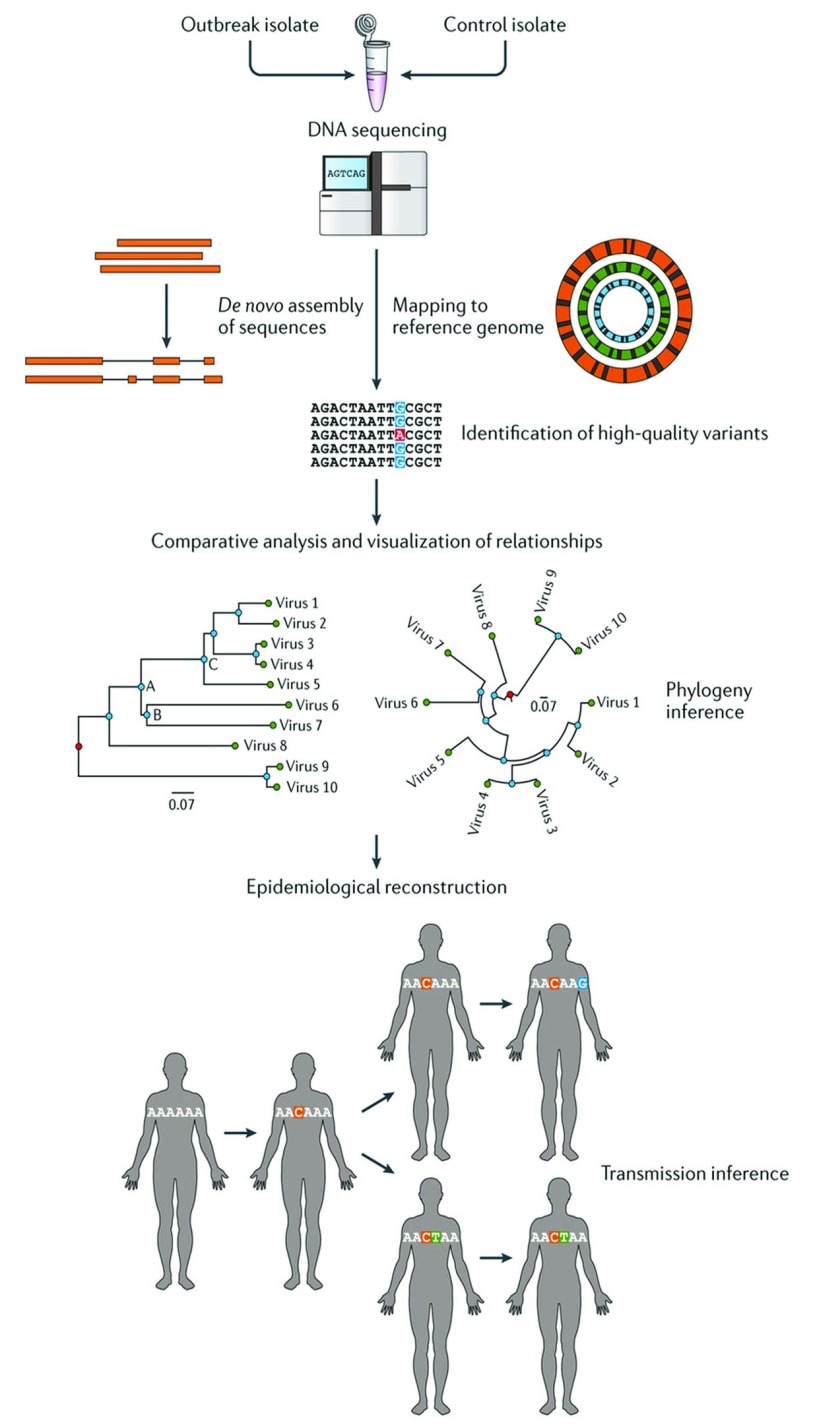
Schematic diagram demonstrating the process and utility of genomic epidemiology. Initially, microbial isolates undergo whole genome sequencing (WGS). WGS can be assembled *de novo* or by mapping to a reference. Bioinformatics platforms enable the uploaded WGS to be annotated and allow users (microbiologists, bioinformaticians, public health officials, and clinicians) to analyse the genes of interest by visualising phylogenetic relationships and associating these with appropriate and relevant meta data. The example of outbreak tracing is used here but this can be extrapolated to many areas of health and disease. Figure reproduced unchanged with permission ^12^.

Genomic epidemiology aims to achieve “systematic investigation of how natural genomic variation affects the clinical outcome of disease” ^11^. The utility of this methodology in the prevention of bacterial meningitis lies in understanding transmission networks, population structure of bacterial pathogens, and epidemiology. In combination, this can inform vaccine development, implementation, and post-vaccine surveillance ( Figure 3) ^12^.

### Next-generation sequencing technology

Genomic epidemiological studies are increasingly available because of the generation of high-quality microbial genomes with benchtop sequencers, including Illumina, Ion Torrent, and Pacific Biosciences platforms. Portable sequencing devices, such as the Oxford Nanopore MinION, have been used in the field for Ebola and Zika virus epidemics, although at the time of writing they still had higher error rates than other next-generation sequencing technologies, of which the Illumina platform was predominant ^13, 14^. The multiplicity of platforms provides flexibility in the face of diverse scale, research or clinical questions, and settings. The cost of sequencing genomes fell rapidly since its commercial inception, but the challenge remains in developing bioinformatics techniques for systematic analyses, which are inexpensive, standardised, highly reproducible worldwide, and easily accessible to microbiologists, epidemiologists, and clinicians alike ^15^.

### Bioinformatics approaches

The most widely used sequence-based method for typing bacteria is MLST, which uses housekeeping genes to catalogue diversity and has been successful because of its highly discriminatory, portable, and unambiguous results ^16^. Genetic lineages within bacterial populations are still most frequently defined by MLST, even when whole genome sequencing (WGS) data are available. By assigning unique alleles at each locus, irrespective of whether the alleles have arisen by individual mutations or HGT, one can systematically index genetic diversity, regardless of the rates of vertical or horizontal transmission ^16^. However, the resolution attainable by seven MLST loci is limited by the small number of loci indexed ( Figure 2a). This can be overcome by using the hierarchical and scalable gene-by-gene approach with assembled WGS data ^20^. By following the principles of MLST but employing more loci, for example, in ribosomal MLST, core genome or whole genome MLST, one can successively increase resolution to identify genetic diversity ( Figure 2b). Typing at other loci allows characterisation of potential vaccine components or virulence factors, including capsule loci, outer membrane proteins, and antibiotic resistance-encoding genes. Alternative approaches include single-nucleotide polymorphism (SNP) typing, where short-read data are mapped onto a reference sequence, after which the SNPs can be identified (SNP calling). The SNPs are collated to reconstruct a phylogeny or into an SNP address, identifying closely related isolate clusters within a given isolate collection, which can be interpreted with additional epidemiological data. This method can be performed rapidly, easily, and sensitively but is dependent on specialist software, reference genomes and sequencing platforms, which can limit portability among sites ^21^.

Effective bioinformatics platforms are required to enable the storage and analysis of WGS data. Databases that allow sharing of WGS include the publicly available Bacterial Isolate Genome Sequence Database (BIGSdb) platform (PubMLST.org), which stores assembled and annotated genome data from the meningococcus and pneumococcus ^20, 22^. This supplements resources such as the International Nucleotide Sequence Database Collaboration, which is composed of the National Center for Biotechnology Information, the European Nucleotide Archive and the DNA Databank of Japan. Global surveillance systems using standardised typing methods are beginning to incorporate data derived from sequence-based technology; some examples include the European Centre for Disease Prevention and Control, the European Surveillance System (TESSy) and US Centers for Disease Control and Prevention PulseNet. In the UK, the Meningitis Research Foundation Meningococcus Genome Library (MRF-MGL) is a repository of WGS of all culture-confirmed meningococcal isolates from 2010 onwards, publicly available through PubMLST.org/neisseria ^23^. Here, some examples of how genomic epidemiology has informed preventative strategies for infections caused by *N. meningitidis* will be outlined.

## Genomic epidemiology of *N. meningitidis*

### Transmission dynamics

***Learning from carriage studies.*** As acquisition is the prerequisite of invasive disease, it is crucial to understand the asymptomatic transmission cycle of the meningococcus and the impact of preventative interventions on herd immunity. The UK meningococcal C vaccine programme, introduced in 1999, was successful by inducing both direct and indirect protection, reducing nasopharyngeal carriage of serogroup C and genogroup C meningococci ^24^. This was the first demonstration of how MLST could be employed at scale in carriage studies for pre- and post-vaccine surveillance ^24^. Subsequently, serogroup B disease has persisted, caused by multiple ccs, although rates of disease have been declining naturally since 2000. A large follow-up carriage study of UK adolescents was done in 2014/15 to assess whether this reduction in disease was associated with rates of meningococcal carriage.

**Figure 4.**
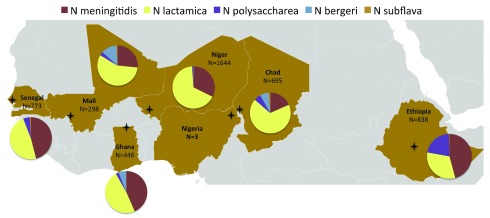
Distribution of *Neisseria* species from pharyngeal carriage across the meningitis belt in sub-Saharan Africa. The proportions of the different species vary markedly in these cross-sectional carriage surveys, which investigated seven countries across the belt. These studies analysed carriage of individuals by age group: 0–4 years, 5–14 years, 15–29 years and 30 or more years. Crosses indicate the sampling area for the respective countries. Figure reproduced unchanged under CC BY ^28^.

***Neisseria species interactions.*** The factors determining progression from nasopharyngeal carriage to invasive disease remain incompletely understood, and it is possible that interactions with other *Neisseria* species are important. To further investigate this area, WGS enabled the development of an accurate, rapid, portable, and affordable method of *Neisseria* species identification. Ribosomal MLST (rMLST) produces highly accurate species and subspecies identification but requires data from 53 loci, optimally generated by WGS ^25^. For use in the MenAfriCar study, WGS data were used to identify a single 413–base pair fragment of the *rplF* gene, the sequence of which generates results consistent with those from rMLST ^26^. The rapid and cost-effective *rplF* assay identified 10.2% carriage of *Neisseria* spp., with point prevalence of *Neisseria lactamica* 5.6%, *N. meningitidis* 3.6%, *Neisseria polysaccharea* 0.6%, *Neisseria bergeri* 0.2% and *Neisseria subflava* 0.05% in the African meningitis belt, which varied markedly by country ( Figure 4) ^27, 28^. *N. lactamica* was carried at the highest rate of 14.1% by 1- to 4-year-olds, and *N. meningitidis* was carried at the highest rate of 5.2% by 5- to 14-year-olds ^28^. Furthermore, there was a mean 4.7-year delay in acquisition of *N. meningitidis* following *N. lactamica* carriage ^29^. Although the underlying mechanisms are yet to be elucidated, this observation has implications for intervention strategies. Since 2010, the PsA-TT vaccine has been progressively implemented across the African meningitis belt, and there has been a dramatic reduction in hyperepidemic meningococcal disease and carriage in vaccinated and unvaccinated individuals ^30^.

***Manipulating the nasopharyngeal niche.*** The age-specific rate of meningococcal carriage and invasive disease is inversely proportional to the rate of colonisation with harmless *N. lactamica*, and alternative prophylactic strategies that exploit this observation have been proposed ^31– 33^. For example, nasal inoculation with live *N. lactamica* has been investigated in UK university students. New colonisation with *N. lactamica* occurred from two weeks after inoculation, and carriage of meningococci fell from 24.2% (n = 36/149) to 14.7% (n = 21/143) ( *P* = 0.006) in those individuals carrying *N. lactamica* ^34^. This effect may be due to displacement of resident *N. meningitidis* soon after colonisation with *N. lactamica*, or in those not colonised with either *Neisseria* spp. at baseline, the colonisation with *N. lactamica* might inhibit meningococcal acquisition. There remained a group of study participants persistently colonised with *N. meningitidis* despite *N. lactamica* challenge, suggesting that displacement can be inhibited. The serogroup distribution was not characterised, so it is not known whether this effect was related to all serogroups; however, the effect was seen across ccs ^34^.

### Extent of capsular group B vaccine coverage

***Estimating protein-based ‘serogroup B substitute’ vaccine coverage.*** The serogroup B polysaccharide capsule is poorly immunogenic and shows structural similarity to human tissue, raising safety concerns. Protein-based ‘serogroup B substitute’ vaccines, including 4CMenB (Bexsero ^®^, GlaxoSmithKline) and bivalent rLP2086 (Trumenba ^®^, Pfizer), were developed to address this issue ^35, 36^. In September 2015, 4CMenB vaccine Bexsero ^®^ was introduced for infants in the UK immunisation schedule at 2, 4, and 12 months of age. This vaccine contains multiple subcapsular proteins, including factor H–binding protein (fHbp), Neisserial heparin binding antigen (NHBA), *Neisseria* adhesin A (NadA), and an outer membrane vesicle (OMV) containing Porin A (PorA). Efficacy of these vaccines must be considered in terms of host immunogenicity and strain coverage. Owing to the practical constraints of performing multiple serum bactericidal assays (SBAs), the accepted correlate of protection for meningococcal vaccines, alternative assays were devised to estimate coverage. Meningococcal strain coverage estimates for England and Wales were 73% (95% confidence interval [CI] 57–87%) using the Meningococcal Antigen Typing System (MATS) ^37^. MATS assesses potential immunological cross-reaction of meningococcal isolates but (i) can be performed by specialist laboratories only, (ii) is expensive and time- and labour-intensive, and (iii) relies on pooled infant serum. Genomic analysis can be used to measure vaccine antigen prevalence using Bexsero ^®^ Antigen Sequence Typing (BAST), implemented on PubMLST.org/neisseria ^38^. Analysis of a collection of 2016 UK isolates, comprising all serogroups from 2010/11 and 2013/14, estimated coverage between 22.8 and 30.8%, increasing to 58.3–60.3% when potentially cross-reactive antigens were included ^38, 39^. A genotype-phenotype association using the seven MLST loci to predict MATS coverage of serogroup B isolates estimated 66% coverage, the same as a subsequent revision of MATS coverage for contemporaneous isolates (2014/15) ^38, 39^.

Estimating the breadth of coverage is equally problematic for the other licenced vaccine, bivalent rLP2086. The Meningococcal Antigen Surface Expression (MEASURE) assay was established to estimate potential coverage by using fHbp surface expression levels, measured by flow cytometry with monoclonal antibody binding to conserved epitopes of fHbp found in both subfamilies contained in the vaccine ^40^. Surface expression had previously been identified as the best predictor of susceptibility of strains in SBAs ^36^. A limitation of both MATS and MEASURE assays is the diversity of disease-causing meningococci. However, this can be appreciated with genomic analysis using large databases such as PubMLST.org/neisseria, and publicly available tools can be used to identify the presence of bivalent rLP2086 fHbp variants and cross-reactive antigens in regions where vaccines are being assessed for implementation or in outbreak settings.

***Pre- and post-vaccine surveillance.*** A pre-vaccine genomic surveillance study identified the prevalence of Bexsero ^®^ antigenic variants to be very low among 3,073 UK disease isolates: fHbp 1, 13.4%; NHBA 2, 13.8%; NadA 8, 0.8%; and PorA-VR P1.4, 10.9% ^41^. This suggested that if the vaccine is to be effective, it would need to be through cross-protective immune responses or through alternative mechanisms involving other OMV proteins, which are poorly understood to date. Consequently, ongoing surveillance is necessary to monitor the secular changes in cc distribution that underlie changes in antigenic variant prevalence, to inform possible vaccine reformulation.

Ten months after vaccine implementation, Bexsero ^®^ efficacy was estimated at 82.9% with very wide CIs (95% CI 24.1–95.2) ^42^. Two years after implementation, disease in the cohort of infants vaccinated with high uptake (92.6%) in 2016/17 represented 12% of the serogroup B burden compared with 19% in 2015/16, when they would not have been fully protected, and 24% in 2014/15 pre-vaccine implementation ^43^. At that time, it was difficult to disentangle vaccine benefit from natural variation in disease rate as serogroup B, C, and Y disease all decreased in 2016/17 compared with 2015/16 on an overall downward trend for over a decade ^43^.

***Capsular group B vaccines and carriage.*** Owing to its uncertain impact on carriage, Bexsero ^®^ has been limited to infants in the UK and Ireland national immunisation programmes. If meningococcal carriage were eliminated by vaccination, herd immunity could prevent disease in other age groups, improving vaccine cost-effectiveness. Observations from a US university outbreak suggested that vaccination did not eliminate carriage. A close contact of individuals who had been vaccinated with two doses of Bexsero ^®^ acquired the same outbreak strain and died; however, such anecdotal data cannot be regarded as conclusive ^44^. The largest study to date, of 2,968 UK university students, comparing Bexsero ^®^ with ACWY vaccine and unvaccinated controls, demonstrated a modest effect of Bexsero ^®^ on carriage of all *N. meningitidis* with 18.2% (95% CI 3.4–30.8) reduction and on serogroup B disease-associated STs with 12.6% (95% CI −15.9–34.1) reduction at least three months after vaccination ^45^. At the time of writing, multi-centre carriage studies were under way in South Australia and the UK to address the impact of protein vaccines on meningococcal carriage. Genomic techniques were being used to capture population structure of carried meningococci in adolescents before and after targeted vaccination with Bexsero ^®^ and Trumenba ^®^.

### Emergence of new strains

**Figure 5.**
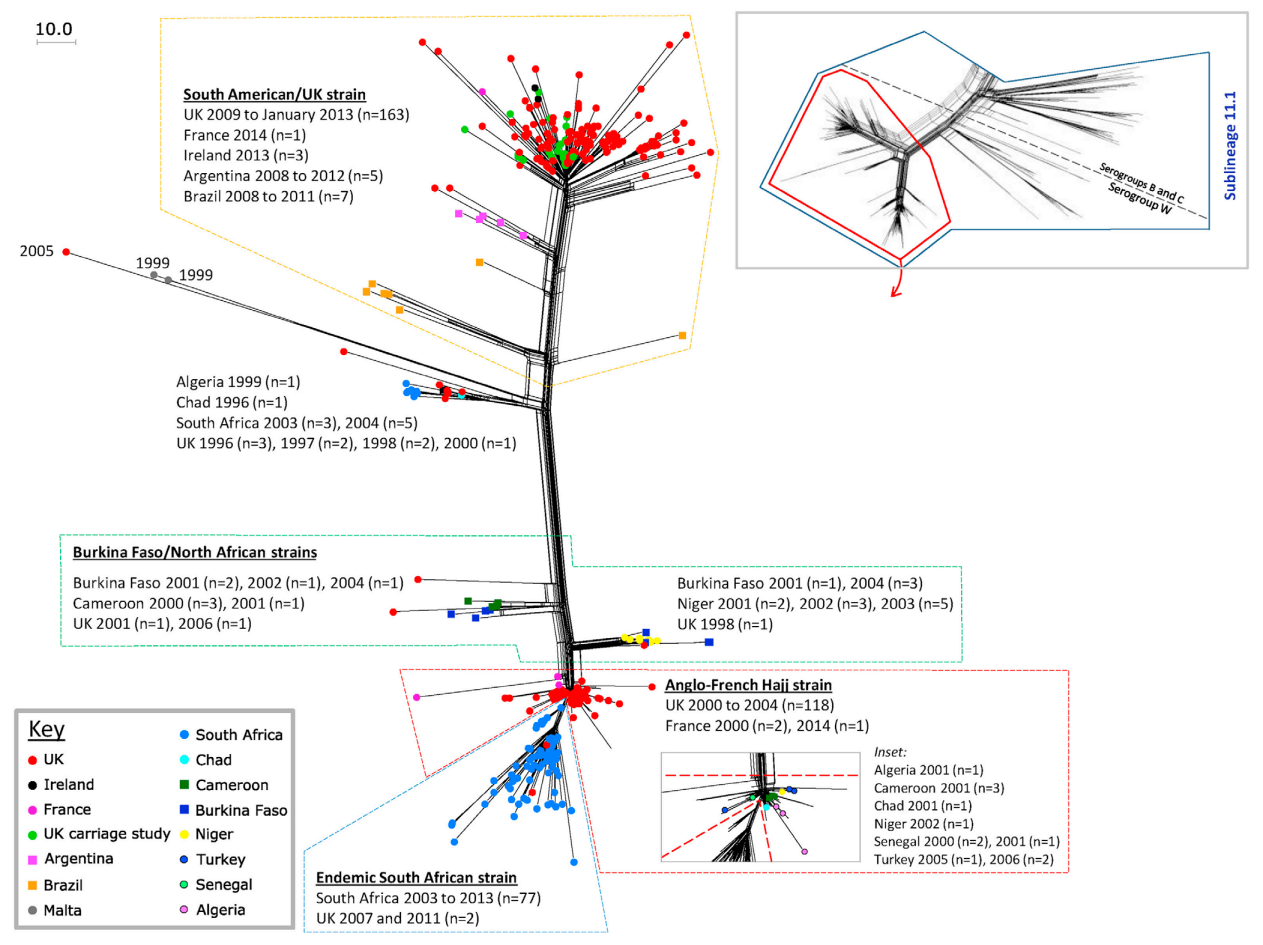
Geotemporal distribution of lineage 11.1 serogroup W isolates in global collections. The inset panel shows a Neighbour-net phylogenetic network of sublineage 11.1 and the distribution of capsular groups within it. Global disease isolates with serogroup W capsular antigens have been analysed by using the meningococcal core genome (cgMLST, consisting of 1,546 loci) and visualised with a Neighbour-net network in the main figure, allowing high-resolution discrimination between isolates. The South American/UK strain has been expanding since 2012 and is seen to be distinct from the Hajj strain. Figure reproduced unchanged under CC BY ^47^.

***Expansion of serogroup W South American/UK strain.*** Serogroup W, cc11 disease outbreaks were first reported in 2000, associated with the Hajj pilgrimage, but following targeted vaccination programmes, disease decreased ^46^. Since 2009–10, however, there was a steady increase in serogroup W cases globally. Genomic epidemiological studies identified the organism responsible as belonging to lineage 11.1 but distinct from a closely related strain associated with the Hajj pilgrimage. This epidemic strain was first seen in South America and subsequently spread worldwide ( Figure 5) ^47^. WGS analysis of MRF-MGL isolates showed that most UK disease after 2013 was due to a new sub-strain varying by only 30 loci, likely due to HGT, indicating microevolution of the aggressive genotype ^48^. The disease caused by this strain has been particularly severe, affecting all age groups with atypical manifestations, including gastrointestinal symptoms, pneumonia, septic arthritis, and epiglottitis/supraglottitis in addition to septicaemia and meningitis ^49– 51^. In response to this outbreak, conjugate meningococcal ACWY vaccine was introduced into the UK immunisation schedule in August 2015 for adolescents, historically those with the highest carriage ^49, 52^. A modest reduction in carriage of serogroups C, W, and Y—from 36.2 to 33% (CI 15.6–51.7)—was seen at least two months after conjugate ACWY vaccination amongst UK university students ^45^. Although this effect on carriage is relatively limited, this may impact on disease incidence due to reduction in acquisition rates.

## Conclusions

Genomic epidemiology of disease-causing bacteria has far-reaching implications for promoting human health and preventing disease. The ability to perform such studies has been accelerated with increasing ease, rapidity, and affordability of WGS. This simultaneously presents challenges to develop methods for distributing and analysing these data for non-specialists. In the case of bacterial meningitis and related diseases, *S. pneumoniae* and *N. meningitidis* have been extensively studied by WGS, and studies of the meningococcus have increased our understanding of carriage, transmission, interactions with commensal *Neisseria* and the distribution of vaccine antigens in national surveillance and emergent organisms. This information has helped to shape vaccination strategies worldwide, ultimately reducing the burden of this devastating disease.
